# Supplementary material for: Hydrothermal plumes as hotspots for deep-ocean heterotrophic microbial biomass production
Source: Nat Commun. 2021 Nov 25;12:6861. doi: 10.1038/s41467-021-26877-6 (PMC8617075; doi:10.1038/s41467-021-26877-6)
Supplement: Supplementary file 1 — Supplementary information [file 41467_2021_26877_MOESM1_ESM.pdf]

CATHALOT et al, 2020

Hydrothermal plumes as hotspots for deep-ocean heterotrophic microbial biomass production - Supplementary information

Contents:

- Tables:

Table S.1. Chemical and physical parameters of our hydrodynamic and microbial model. Initial values for high temperature fluids for each study sites are listed.

Table S.2. Model parameters

Table S.3. Error assessment of our hydrodynamic model on the NBP substrate concentrations, and associated biomass production rates

- Figures:

Figure S.1: Plume schematics used in the hydrodynamic model

Figure S.2. Sensitivity analysis performed on the Crab Vent field site. Influence of the V_{harv} parameter ranging from 1000 to 10000.

Figure S.3. 300 days model runs for the Endeavour Main Field and TAG vent sites. Top: model outputs for biomass production rates for the different metabolic groups. Bottom: model output for activity rates for the different metabolic groups.

- Supplementary References

Table S. 1. Chemical and physical parameters of our hydrodynamic and microbial model. Initial values for high temperature fluids for each study sites are listed.

Temp : temperature. DOC_l : labile DOC, DOC_r : refractory DOC

	**TAG[a,b]**	**Rainbow[c]**	**Tour Eiffel[d]**	**Broken Spur[c]**	**Ashadze[c]**	**Logatchev[c]**	**EPR Grand Bonum[c]**	**Dante[c]**	**Kairei[c]**	**Edmonds[c]**	**Crab Spa**	**Seawater**
Chemical parameters												
**pH**	2.83	3.10	3.75	4.00	3.10	3.30	3.30	4.25	3.35	4.81	5.6	7.9
Species (mM)												
**ΣH_2S**	3.600	1.200	2.100	9.750	1.000	1.400	4.500	4.000	4.000	4.810	0.552	0
**S**	0	0	0	0	0	0	0	0	0	0	0	0
**$S_2O_3^{2-}$**	0	0	0	0	0	0	0	0	0	0	0	0
**SO_4^{2-}**	0	0.6	0	0	0	0	0	0	0	0	25.8	28.9
**NH_4+**	0	4.1	0	0	0	0	0.01	0.453	0.038	0.040	0.012	10^{-5}
**NO_2^-**	0	0	0	0	0	0	0	0	0	0	0	10^{-7}
**NO_3^-**	0	0	0	0	0	0	0	0	0	0	0.132	18.1
**Fe^{2+}**	1.640	25.000	0.624	2.000	9.300	2.500	9.856	0.443	6.010	13.900	0	10^{-7}
**Mn^{2+}**	1.000	22.900	0.289	0.255	1.062	0.367	1.241	0.189	0.857	1.430	0	$2.5.10^{-7}$
**Mg^+**	0	0	0	0	0	0	0	0	0	0	0	52.7
**FeOOH**	0	0	0	0	0	0	2.10^{-6}	0	0	0	0	0
**HCO_3^-**	$3.4.10^{-6}$	$2.8.10^{-5}$	$4.9.10^{-5}$	$2.0.10^{-5}$	$3.8.10^{-6}$	$4.2.10^{-6}$	$5.9.10^{-5}$	$1.4.10^{-4}$	$8.2.10^{-6}$	$5.1.10^{-4}$		$2.2.10^{-3}$ – $1.6.10^{-5}$
**CO_2**	4.100	16.000	23.000	6.550	3.700	4.400	29.000	13.100	5.130	7.190	8.200	17.10^{-3}
**CH_4**	0.118	2.200	0.680	0.098	1.200	2.600	37.000	0.006	0.203	0.289	0.008	$2.5.10^{-7}$
**H_2**	0.075	16.000	0.122	0.730	19	12.500	0.100	0.029	8.190	0.142	0.179	3.10^{-7}
**O_2**	0	0	0	0	0	0	0	0.004	0	0	0.107	0.267
**N_2**	0.900	0.900	1.580	0.900	1.580	5.500	1.580	1580	1.580	1.58	1.000	0.59
**DOC_l**	0.500	0.100	0.200	0.200	0.200	0.147	0.140	0.140	0.200	0.200	0.100	10^{-5}
**DOC_r**	0	0	0	0	0	0	0	0	0	0	0	0.044
**POC**	0	0	0	0	0	0.023	0.023	0.023	0.023	0.023	0.023	3.10^{-4}
Physical parameters												
**Temp (°C)**	362	365	324	364	352	350	350	334	365	-	-	2-3.3
**Salinity (‰)**	42	44	24.7	27.7	36.2	35	38.536	30.3	36.2	-	-	31.8-34.946

**Vent exit diameter (m)**	0.18	0.12	0.057	0.05	0.2	0.06	0.033	0.056	0.05	-	-	-
**Vent velocity (m/s)**	0.5	39.8	1.19	1.5	2	1.5	0.63	0.916	0.5	-	-	-

Data for the TAG vent field come from a) the BICOSE cruise and b)[1]. c) Data for the Rainbow and BrokenSpur vent field issued from[2,3], for Tour Eiffel from[4], for East Pacific Rise Grand Bonum from[5], for Ashadze from[1], for Dante from[6], for Kairei from[7], Edmonds from[7].

Vent diameters and velocities were collected from literature[8-10]. For the Kairei and Broken Spur vent field, they were hypothesized based on the volume flux estimates available[11,12].

When physical parameters were not available a 10^5 dilution factor was applied according to[13].

DOC concentrations in TAG fluids were measured from samples collected during the BICOSE and BICOSE2 cruises[14]. DOC concentrations in hydrothermal fluids from the Logatchev vent sites are set according to[15]. Since no actual data from the Rainbow vent site were available, DOC concentrations of the Rainbow fluids were set according to 100µM, similar to the ultramafic Lost City site as measured by[16]. All other vent sites along the Mid-Atlantic Ridge, and at the Kairei and Edmonds vent fields were set to 200 µM[15,17]. DOC concentrations at the Crab Spa vent site were assumed to be lower[18,19] and set to 100µM, while DOC concentrations at the Dante and Grand Bonum site were assumed to be around 140µM[17,20]. All DOC in the hydrothermal fluid were assumed to be labile DOC, as high volatiles and bioavailable compounds concentrations have been documented in hydrothermal fluids[14,16,18]. Seawater DOC concentration was taken from [16] and assumed to be mostly refractory[21,22], with only 0.01µM of labile DOC[23].

Table S.2. Model parameters

**Microbial parameters**	
Available volume per cell for substrate assimilation (V_{harv})	1000 m^3 $molBiomass^{-1}$
Background Cells concentration[a]	10^{11} cell m^{-3}
Cell volume	10^{-18} m^3
Cell C content	141.10^{-15} gC
Biomass Molecular weight	24.68 g $molBiomass^{-1}$
Biomass C content	0.48 gC.$gBiomass^{-1}$
Mortality rate (α_m)[b]	1.16 10^{-8} s^{-1}
Internal recycling from mortality	40% in labile DOC (DOC_l)<br>10% in refractory DOC (DOC_r)<br>50% in POC
**Thermodynamic parameters**	
Gas constant R	8.31446 J K^{-1} mol^{-1}
Boltzmann constant	1.38065 10^{-23} J K^{-1}
Planck constant	6.62607 10^{-34} J.s
Universal gravitational constant g	9.80665 m.s^{-2}
**Hydrodynamic parameters**	
Entrainment coefficient for pure jet α_j	0.06
Entrainment coefficient for pure buoyant plume α_p	0.16
Froude number for pure buoyant plume Fr_p	1.6

[a] Based on BICOSE2 cruise measurements

[b] Based on [24]

Table S.3. Error assessment of our hydrodynamic model on the NBP susbtrate concentrations, and associated biomass production rates

			Non-Buoyant Plume (NBP) concentrations (nM)								Biomass production (µgC l^{-1} d^{-1})	
**Site**	**Type of data**	**Ref**	**CH_4**	**Mn**	**Fe**	**H_2**	**H_2S**	**NH_4**	**NO_3^-**	**NO_2^-**	**HP**	**PP**
**TAG**	*data*	25-27	0.6-18	11-82	51	*n.a.*	*n.a.*	*n.a.*	*n.a.*	*n.a.*	*NBP model output similar to data*	
	model outputs		2.1	17.5	28.4	1.6	62.1	100.0	18.1 10^3	0.1		
**Edmonds**	*data*	28	*n.a.*	*n.a.*	200-600	*n.a.*	*n.a.*	*n.a.*	*n.a.*	*n.a.*	*NBP model output similar to data*	
	model outputs		3.1	14.6	139.1	301.4	48.1	100.4	18.1 10^3	0.1		
**Kairei**	*data*	28,29	10-40	*n.a.*	100-450	*n.a.*	*n.a.*	*n.a.*	*n.a.*	*n.a.*	*NBP model output similar to data*	
	data	30	0.6-3	2-12	6-40	*n.a.*	*n.a.*	*n.a.*	*n.a.*	*n.a.*		
	model outputs		1.6	5.9	39.8	354.1	26.4	100.2	18.1 10^3	0.1		
**Endeavour Dante**	*data*	31 32-34	20-600	10-50; 80	50-250	*n.a.*	*n.a.*	200-400	*n.a.*	*n.a.*	*NBP model output similar to data*	
	model outputs		600	100	100	0.3	0	400	18.1 10^3	0.1		
**Rainbow**	*data*	10,35	160	160	1200	*n.a.*	2	*n.a.*	*n.a.*	*n.a.*	0.0124	0.0000
	model outputs		216	2245.9	2451.6	1569.3	117.7	502	18.1 10^3	0.1	0.0124	0.0074
**Logatchev**	*data*	36-38	14-350	100	230	1600	*n.a.*	*n.a.*	*n.a.*	*n.a.*	0.0092	0.0000
	model outputs		30.3	4.5	29	144.7	16.2	100	18.1 10^3	0.1	0.0092	0.0000
**Broken Spur**	*data*	39,40	*n.a.*	2-11	15-35	*n.a.*	*n.a.*	*n.a.*	17 10^3	20	0.0088	0.0000
	model outputs		0.4	0.7	3.6	1.6	16.9	100	18.1 10^3	0.1	0.0088	0.0000

n.a.: not available

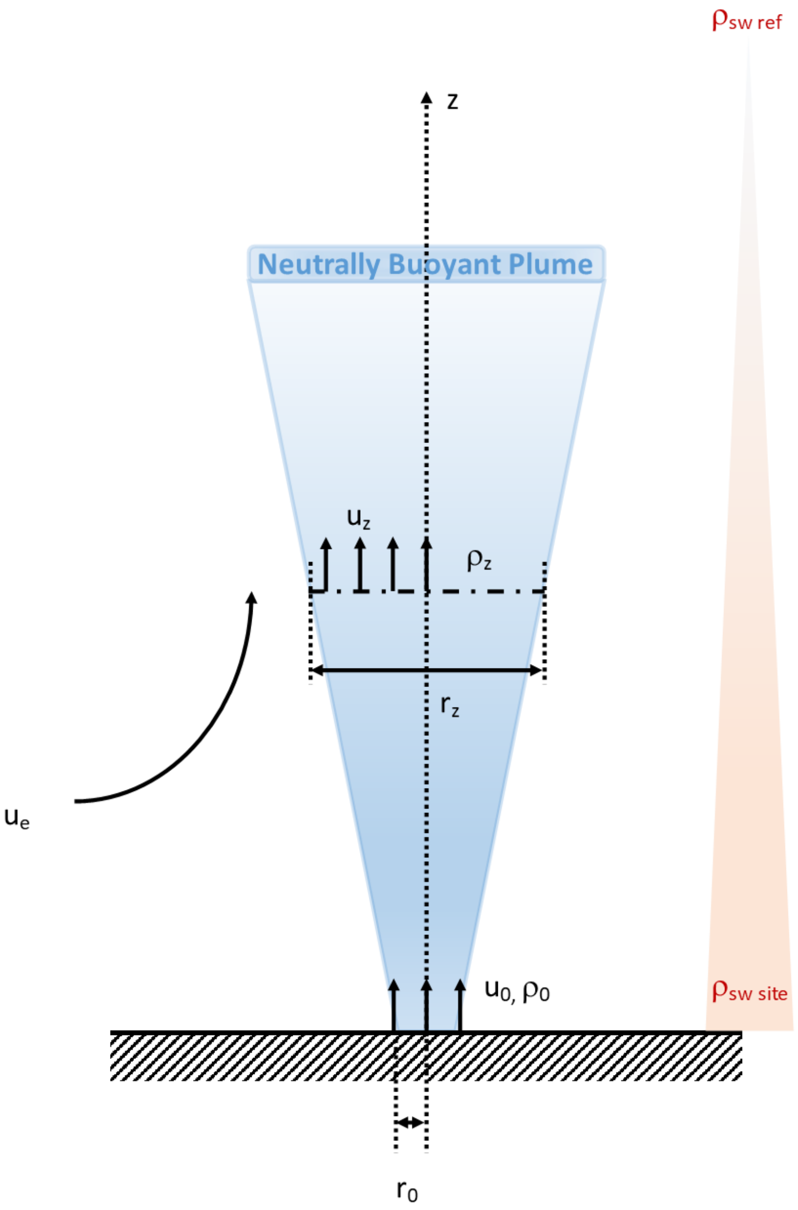

Figure S.1. Plume schematics used in the hydrodynamic model

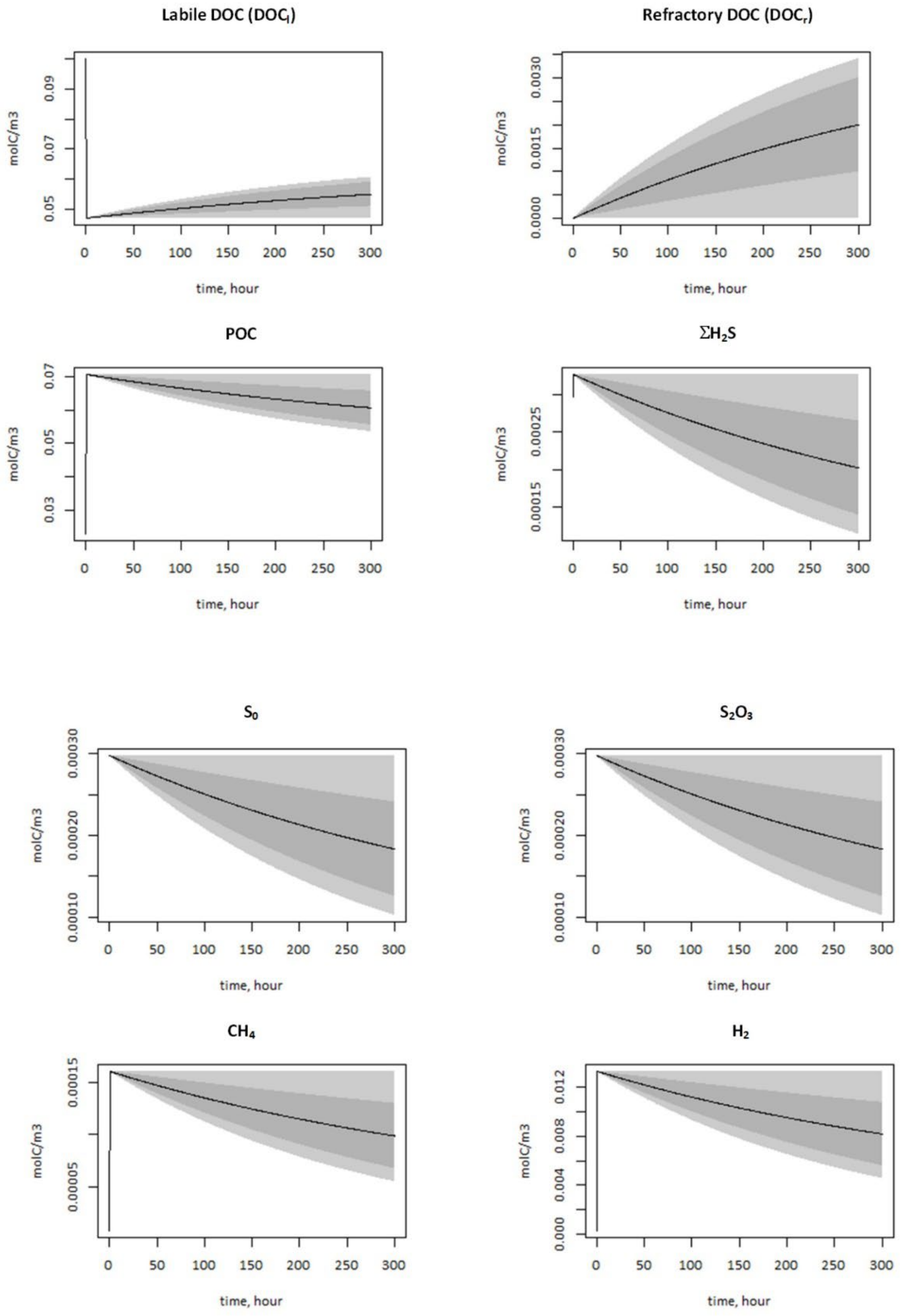

Figure S.2. Sensitivity analysis performed on the Crab Vent field site. Influence of the V_{harv} parameter ranging from 1000 to 10000. Center line represents the average output for the sensitivity analysis, dark grey envelope represents the mean +/- standard deviation of the outputs and the light grey envelope represents the minimum and maximum outputs obtained for the sensitivity analysis.

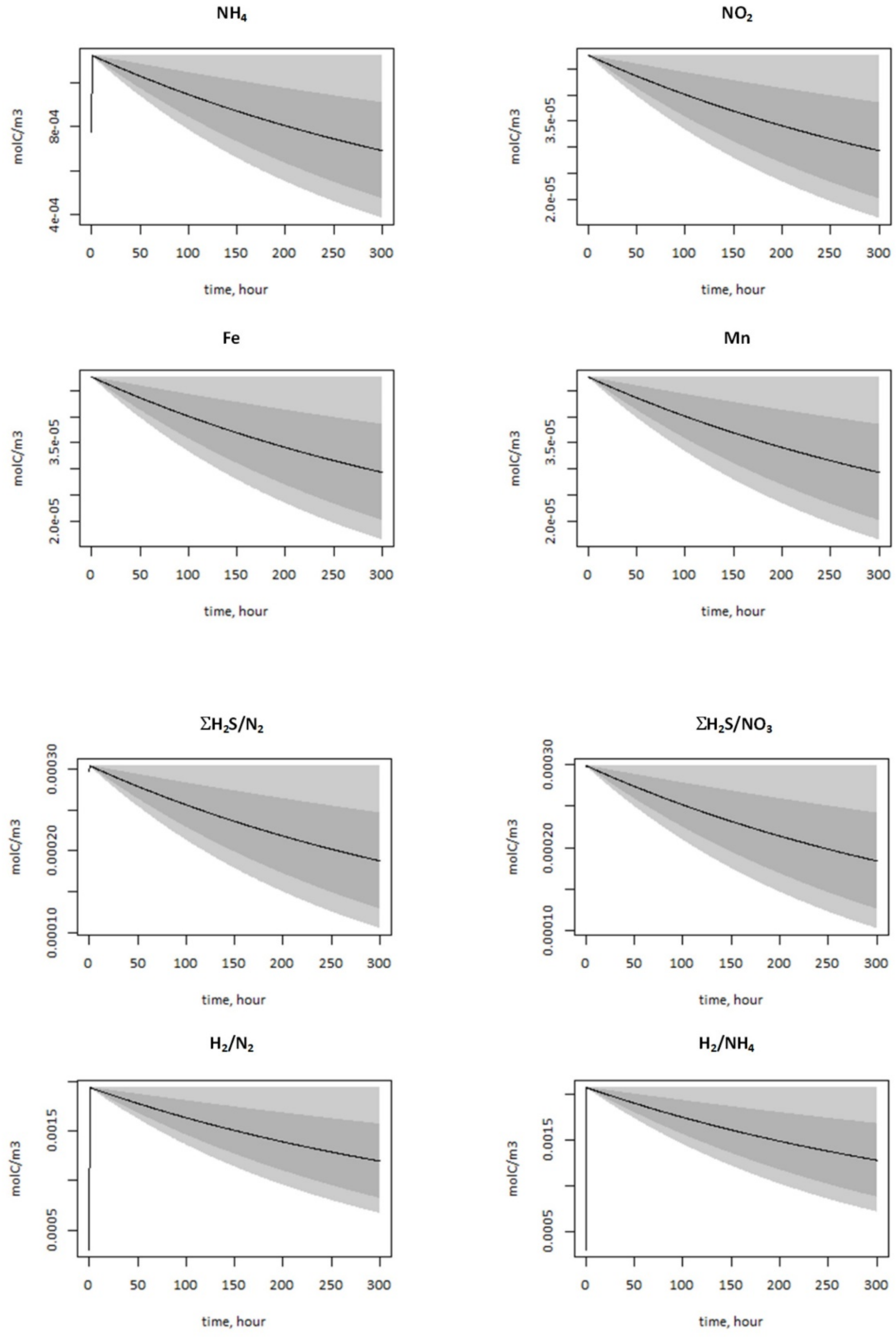

Figure S.2. (continued)

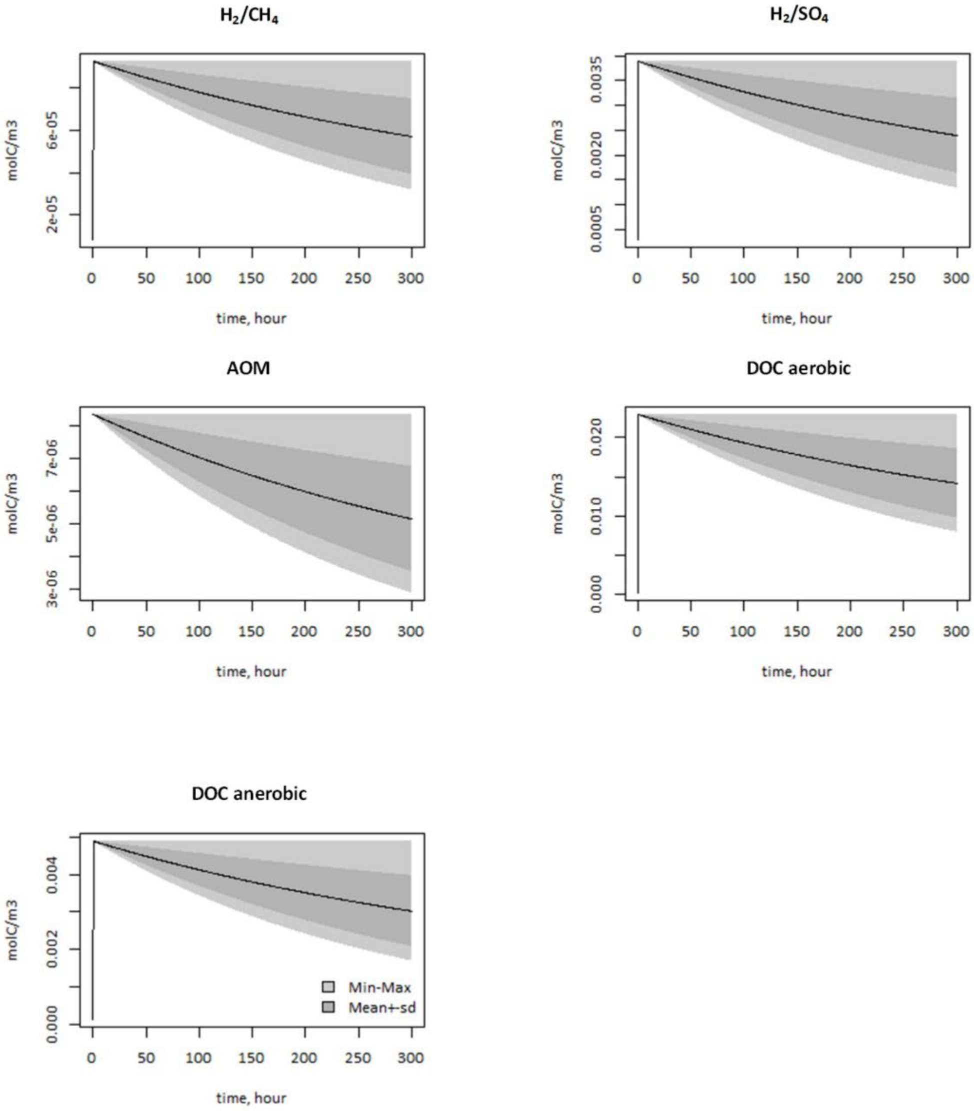

Figure S.2. (continued)

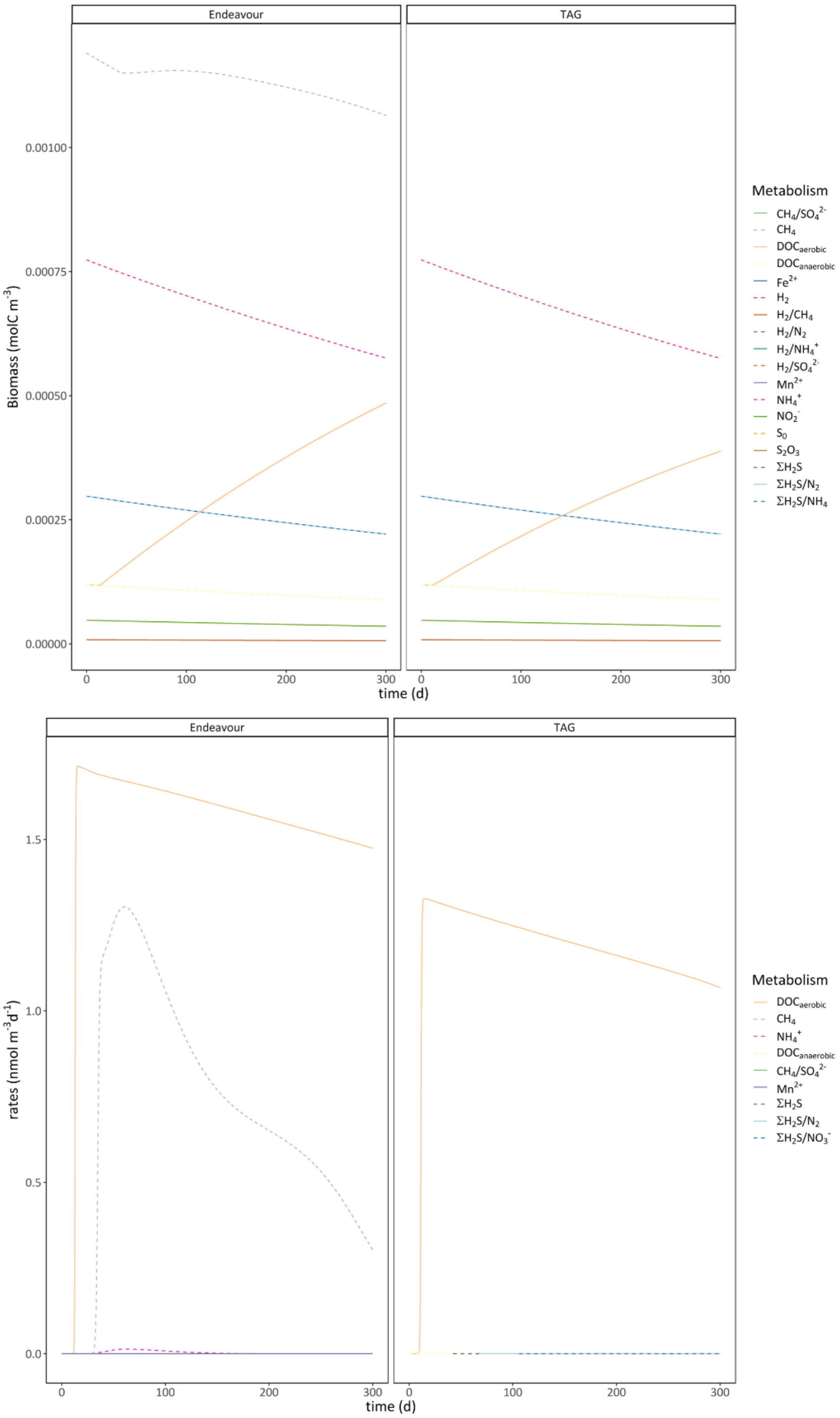

Figure S.3. 300 days model runs for the Endeavour Main Field and TAG vent sites. Top: model outputs for biomass production rates for the different metabolic groups. Bottom: model output for activity rates for the different metabolic groups.

Supplementary references:

1 Charlou, J. L. *et al.* in *Diversity of Hydrothermal Systems on Slow Spreading Ocean Ridges* Vol. 188 *Geophys. Monogr. Ser.* 265-296 (AGU, 2010).

2 Charlou, J. L., Donval, J. P., Fouquet, Y., Jean-Baptiste, P. & Holm, N. Geochemistry of high H(2) and CH(4) vent fluids issuing from ultramafic rocks at the Rainbow hydrothermal field (36 degrees 14 ' N, MAR). *Chemical Geology* **191**, 345-359, doi:10.1016/s0009-2541(02)00134-1 (2002).

3 James, R. H., Elderfield, H. & Palmer, M. R. The chemistry of hydrothermal fluids from the Broken Spur site, 29°N Mid-Atlantic ridge. *Geochim. Cosmochim. Acta* **59**, 651-659, doi:https://doi.org/10.1016/0016-7037(95)00003-I (1995).

4 Charlou, J. L. *et al.* Compared geochemical signatures and the evolution of Menez Gwen (37°50′N) and Lucky Strike (37°17′N) hydrothermal fluids, south of the Azores Triple Junction on the Mid-Atlantic Ridge. *Chemical Geology* **171**, 49-75, doi:http://dx.doi.org/10.1016/S0009-2541(00)00244-8 (2000).

5 Pester, N. J., Rough, M., Ding, K. & Seyfried, W. E. A new Fe/Mn geothermometer for hydrothermal systems: Implications for high-salinity fluids at 13°N on the East Pacific Rise. *Geochim. Cosmochim. Acta* **75**, 7881-7892, doi:https://doi.org/10.1016/j.gca.2011.08.043 (2011).

6 Lin, T. J. *et al.* Linkages between mineralogy, fluid chemistry, and microbial communities within hydrothermal chimneys from the Endeavour Segment, Juan de Fuca Ridge. *Geochemistry, Geophysics, Geosystems* **17**, 300-323, doi:10.1002/2015GC006091 (2016).

7 Gallant, R. M. & Von Damm, K. L. Geochemical controls on hydrothermal fluids from the Kairei and Edmond Vent Fields, 23°–25°S, Central Indian Ridge. *Geochemistry, Geophysics, Geosystems* **7**, doi:https://doi.org/10.1029/2005GC001067 (2006).

8 Germanovich, L. N., Hurt, R. S., Smith, J. E., Genc, G. & Lowell, R. P. Measuring fluid flow and heat output in seafloor hydrothermal environments. *Journal of Geophysical Research: Solid Earth* **120**, 8031-8055, doi:10.1002/2015JB012245 (2015).

9 Mittelstaedt, E. *et al.* Quantifying diffuse and discrete venting at the Tour Eiffel vent site, Lucky Strike hydrothermal field. *Geochemistry, Geophysics, Geosystems* **13**, Q04008, doi:10.1029/2011GC003991 (2012).

10 German, C. R. *et al.* Diverse styles of submarine venting on the ultraslow spreading Mid-Cayman Rise. *Proceedings of the National Academy of Sciences of the United States of America* **107**, 14020-14025, doi:10.1073/pnas.1009205107 (2010).

11 Rudnicki, M. D. & German, C. R. Temporal variability of the hydrothermal plume above the Kairei vent field, 25°S, Central Indian Ridge. *Geochemistry, Geophysics, Geosystems* **3**, XXX-XXX, doi:https://doi.org/10.1029/2001GC000240 (2002).

12 Murton, B. J., Redbourn, L. J., German, C. R. & Baker, E. T. Sources and fluxes of hydrothermal heat, chemicals and biology within a segment of the Mid-Atlantic Ridge. *Earth and Planetary Science Letters* **171**, 301-317, doi:https://doi.org/10.1016/S0012-821X(99)00157-0 (1999).

13 Baker, E. T., German, C. R. & Elderfield, H. in *Seafloor Hydrothermal Systems: Physical, Chemical, Biological, and Geological Interactions* Vol. 91 *Geophys. Monogr. Ser.* 47-71 (AGU, 1995).

14 Konn, C. *et al.* Extending the dataset of fluid geochemistry in hydrothermal vents on the Mid-Atlantic Ridge (23-37°N): time series and organic composition. *Deep Sea Research Part A. Oceanographic Research Papers* (accepted with major revisions).

15 Rossel, P. E. *et al.* Thermally altered marine dissolved organic matter in hydrothermal fluids. *Organic Geochemistry* **110**, 73-86, doi:https://doi.org/10.1016/j.orggeochem.2017.05.003 (2017).

16 Lang, S. Q., Butterfield, D. A., Schulte, M., Kelley, D. S. & Lilley, M. D. Elevated concentrations of formate, acetate and dissolved organic carbon found at the Lost City hydrothermal field. *Geochim. Cosmochim. Acta* **74**, 941-952, doi:10.1016/j.gca.2009.10.045 (2010).

17 Hawkes, J. A. *et al.* Efficient removal of recalcitrant deep-ocean dissolved organic matter during hydrothermal circulation. *Nature Geoscience* **8**, 856-860, doi:10.1038/ngeo2543 (2015).

18 Longnecker, K., Sievert, S. M., Sylva, S. P., Seewald, J. S. & Kujawinski, E. B. Dissolved organic carbon compounds in deep-sea hydrothermal vent fluids from the East Pacific Rise at 9°50’N. *Organic Geochemistry* **125**, 41-49, doi:https://doi.org/10.1016/j.orggeochem.2018.08.004 (2018).

19 Lang, S. Q., Butterfield, D. A., Lilley, M. D., Paul Johnson, H. & Hedges, J. I. Dissolved organic carbon in ridge-axis and ridge-flank hydrothermal systems. *Geochim. Cosmochim. Acta* **70**, 3830-3842, doi:https://doi.org/10.1016/j.gca.2006.04.031 (2006).

20 Butterfield, D. A. *et al.* Gradients in the composition of hydrothermal fluids from the Endeavour segment vent field: Phase separation and brine loss. *Journal of Geophysical Research: Solid Earth* **99**, 9561-9583, doi:doi:10.1029/93JB03132 (1994).

21 Williams, P. M. & Druffel, E. R. M. Radiocarbon in dissolved organic matter in the central North Pacific Ocean. *Nature* **330**, 246-248 (1987).

22 Hertkorn, N. *et al.* Characterization of a major refractory component of marine dissolved organic matter. *Geochim. Cosmochim. Acta* **70**, 2990-3010, doi:https://doi.org/10.1016/j.gca.2006.03.021 (2006).

23 Bendtsen, J., Lundsgaard, C., Middelboe, M. & Archer, D. Influence of bacterial uptake on deep-ocean dissolved organic carbon. *Glob. Biogeochem. Cycle* **16**, 74-71-74-12, doi:https://doi.org/10.1029/2002GB001947 (2002).

24 Reed, D. C. *et al.* Predicting the response of the deep-ocean microbiome to geochemical perturbations by hydrothermal vents. *Isme Journal* **9**, 1857-1869, doi:10.1038/ismej.2015.4 (2015).

25 Charlou, J.-L. & Donval, J.-P. Hydrothermal methane venting between 12°N and 26°N along the Mid-Atlantic Ridge. *Journal of Geophysical Research: Solid Earth* **98**, 9625-9642, doi:10.1029/92JB02047 (1993).

26 Rudnicki, M. D. & Elderfield, H. Theory applied to the Mid-Atlantic Ridge hydrothermal plumes: the finite-difference approach. *J. Volcanol. Geotherm. Res.* **50**, doi:10.1016/0377-0273(92)90043-d (1992).

27 González-Santana, D. *et al.* Processes Driving Iron and Manganese Dispersal From the TAG Hydrothermal Plume (Mid-Atlantic Ridge): Results From a GEOTRACES Process Study. *Frontiers in Marine Science* **7**, doi:10.3389/fmars.2020.00568 (2020).

28 Statham, P. J., German, C. R. & Connelly, D. P. Iron (II) distribution and oxidation kinetics in hydrothermal plumes at the Kairei and Edmond vent sites, Indian Ocean. *Earth and Planetary Science Letters* **236**, 588-596, doi:http://dx.doi.org/10.1016/j.epsl.2005.03.008 (2005).

29 Gamo, T. *et al.* Chemical characteristics of newly discovered black smoker fluids and associated hydrothermal plumes at the Rodriguez Triple Junction, Central Indian Ridge. *Earth and Planetary Science Letters* **193**, 371-379, doi:https://doi.org/10.1016/S0012-821X(01)00511-8 (2001).

30 Gamo, T. *et al.* Hydrothermal plumes at the Rodriguez triple junction, Indian ridge. *Earth and Planetary Science Letters* **142**, 261-270, doi:https://doi.org/10.1016/0012-821X(96)00087-8 (1996).

31 Kadko, D. C., Rosenberg, N. D., Lupton, J. E., Collier, R. W. & Lilley, M. D. Chemical reaction rates and entrainment within the Endeavour Ridge hydrothermal plume. *Earth and Planetary Science Letters* **99**, 315-335, doi:https://doi.org/10.1016/0012-821X(90)90137-M (1990).

32 Sharp, J. L. *Manganese and Iron Geochemistry in the Endeavour Ridge: Hydrothermal Plume* Master of Science thesis, Oregon State University, (1991).

33 Lam, P., Cowen, J. P., Popp, B. N. & Jones, R. D. Microbial ammonia oxidation and enhanced nitrogen cycling in the Endeavour hydrothermal plume. *Geochim. Cosmochim. Acta* **72**, 2268-2286, doi:https://doi.org/10.1016/j.gca.2008.01.033 (2008).

34 Cowen, J. P., Wen, X. & Popp, B. N. Methane in aging hydrothermal plumes. *Geochim. Cosmochim. Acta* **66**, 3563-3571, doi:https://doi.org/10.1016/S0016-7037(02)00975-4 (2002).

35 Radford-Knoery, J., German, C. R., Charlou, J.-L., Donval, J.-P. & Fouquet, Y. Distribution and behavior of dissolved hydrogen sulfide in hydrothermal plumes. *Limnol. Oceanogr.* **46**, 461-464, doi:https://doi.org/10.4319/lo.2001.46.2.0461 (2001).

36 Schmidt, K., Koschinsky, A., Garbe-Schönberg, D., de Carvalho, L. M. & Seifert, R. Geochemistry of hydrothermal fluids from the ultramafic-hosted Logatchev hydrothermal field, 15°N on the Mid-Atlantic Ridge: Temporal and spatial investigation. *Chemical Geology* **242**, 1-21, doi:https://doi.org/10.1016/j.chemgeo.2007.01.023 (2007).

37 Keir, R. S., Schmale, O., Seifert, R. & Sültenfuß, J. Isotope fractionation and mixing in methane plumes from the Logatchev hydrothermal field. *Geochemistry, Geophysics, Geosystems* **10**, Q05005, doi:10.1029/2009GC002403 (2009).

38 Zhou, H., Wu, Z., Peng, X., Jiang, L. & Tang, S. Detection of methane plumes in the water column of Logatchev hydrothermal vent field, Mid-Atlantic Ridge. *Chinese Science Bulletin* **52**, 2140-2146, doi:10.1007/s11434-007-0285-y (2007).

39 German, C. R., Rudnicki, M. D. & Klinkhammer, G. P. A segment-scale survey of the Broken Spur hydrothermal plume. *Deep Sea Research Part I: Oceanographic Research Papers* **46**, 701-714, doi:https://doi.org/10.1016/S0967-0637(98)00078-8 (1999).

40 James, R. H. *et al.* Hydrothermal plumes at Broken Spur, 29°N Mid-Atlantic Ridge: chemical and physical characteristics. *Geological Society, London, Special Publications* **87**, 97-110, doi:10.1144/gsl.Sp.1995.087.01.09 (1995).
